# Functional and Structural Reliability of Optic Nerve Head Measurements in Healthy Eyes by Means of Optical Coherence Tomography Angiography

**DOI:** 10.3390/medicina56010044

**Published:** 2020-01-20

**Authors:** Rodolfo Mastropasqua, Rossella D’Aloisio, Luca Agnifili, Eduardo Zuppardi, Guido Di Marzio, Marta Di Nicola, Annamaria Porreca, Daniele Guarini, Michele Totta, Lorenza Brescia, Luca Di Antonio

**Affiliations:** 1 Vitreoretinal Unit, Bristol Eye Hospital, University of Bristol, Bristol BS8 1TH, UK; rodolfo.mastropasqua@gmail.com; 2 Eye Clinic, Polytechnic University of Marche, 60126 Ancona, Italy; 3 Ophthalmology Clinic, Department of Medicine and Science of Ageing, University G. d’Annunzio Chieti-Pescara, 66100 Chieti, Italy; l.agnifili@unich.it (L.A.); e.zuppardi@alice.it (E.Z.); dimarzio61@alice.it (G.D.M.); guarini.daniele@gmail.com (D.G.); michetto135@gmail.com (M.T.); brescia.lorenza@gmail.com (L.B.); monsieurluca@yahoo.com (L.D.A.); 4 Laboratory of Biostatistics, Department of Medical, Oral and Biotechnological Sciences, University G. d’Annunzio Chieti-Pescara, 66100 Chieti, Italy; mdinicola@unich.it; 5 Department of Economic Studies, University G. d’Annunzio Chieti-Pescara, 66100 Chieti, Italy; porreca.annamaria@gmail.com

**Keywords:** optical coherence tomography angiography, optic nerve head perfusion, optic nerve head vessel density, optical microangiography, repeatability of imaging tools

## Abstract

*Background and Objectives*: the aim of the study was to evaluate the repeatability and reproducibility of optical microangiography (OMAG)-based optical coherence tomography angiography (OCTA) in the optic nerve head (ONH) and radial peripapillary capillary (RPC) perfusion assessment of healthy eyes. *Materials and Methods*: in this observational study, a total of 40 healthy subjects underwent ONH evaluation, using an OMAG-based OCTA system at baseline (T_0_), after 30 min (T_1_), and after 7 days (T_2_). The main outcome measures were the vessel density (VD) and flux index (FI) of the RPCs, as well as peri-papillary retinal nerve fibre layer (pRNFL) thickness. The analysis was performed by two observers independently. The coefficient of repeatability (CR), within the subject coefficient of variation (CVw) and intrasession correlation coefficient (ICC), to evaluate intrasession repeatability of measurements was calculated for each observer. *Results*: the high intrasession and intersession repeatability and reproducibility were assessed in the two observers for all three outcome measures. Of note, the CRs for the first and the second observer were 0.011 (95% confidence interval (CI) 0.009–0.014) and 0.016 (95% CI 0.013–0.020) for FI, 0.016 (95% CI 0.013–0.021) and 0.017 (95% CI 0.014–0.021) for VD, and 2.400 (95% CI 1.948–3.092) and 3.732 (95% CI 3.064–4.775) for pRNFL thickness, respectively. The agreement between them was excellent for pRNFL assessment and very good for FI and VD. *Conclusion*: OCTA has a great potential in the accurate assessment of ONH and peri-papillary microcirculation. It allows for repeated and reproducible measurements without multiple scans-related bias, thus guaranteeing an independent operator analysis with good reproducibility and repeatability.

## 1. Introduction

Over the years, several imaging techniques aimed at analysing the vascular networks of the eye have been developed [1,2]. Different systems have been used to evaluate retinal blood flow parameters, starting from the traditional fluorescein angiography to more recent platforms, such as laser doppler flowmeter or doppler optical coherence tomography [1,2]. Nevertheless, they have all shown high range variability between measurements in identifying ocular vessel perfusion [3,4].

To date, optical coherence tomography angiography (OCTA) is considered to be a new paradigm shift for the evaluation of retinal and optic nerve head (ONH) microcirculation, by replacing the more invasive fluorescein angiography in clinical practice, allowing for a thorough visualization and quantification of vessel density of the retina, ONH, and peri-papillary retina [5,6].

An optical microangiography (OMAG)-based optical coherence tomography angiography (OCTA) system has been recently introduced, and has been shown to have excellent repeatability and reproducibility in the analysis of perfusion features of the ONH in healthy eyes [7,8].

Currently, OCTA is being used to study and characterize the vascular networks in different ocular diseases, as well as to find early pathological signs to be identified on scans in clinical practice. Therefore, it is crucial to understand the applicability and accuracy of this diagnostic tool, studying its repeatability and reproducibility in clinical settings. This topic appears especially critical in assessing chronic optic nerve diseases, such as glaucoma, which may require repeated measurements over time [7].

The aim of the current study was to evaluate the functional and structural reliability of ONH-related measurements in healthy eyes by means of OMAG-based OCTA.

## 2. Materials and Methods

In this observational instrument validation study, a total of 40 healthy subjects underwent ONH evaluation using an OMAG-based OCTA system (Angioplex Cirrus 5000HD-OCT, Carl Zeiss Meditec Inc., Dublin, CA, USA) at baseline (T_0_), 30 min after the first evaluation (T_1_), and after 7 days (T_2_). Measurements at T_0_ and T_1_ were performed in the morning, and at T_2_ were assessed in the afternoon.

The study was conducted in accordance with tenets of Declaration of Helsinki and was approved by our Institutional Review Board (Department of Medicine and Science of Ageing, University “G. d’Annunzio” of Chieti-Pescara, ethical code number: ONH19, date of approval: 30 November 2019). Written informed consent was obtained from the subjects after an explanation of the nature and possible consequences of the study. A comprehensive ophthalmic evaluation was made for each subject examined, including best corrected visual acuity (BCVA) using an ETDRS chart, slit-lamp biomicroscopy with dilated fundoscopy using a 90D lens, intra-ocular pressure (IOP) measurement using Goldmann applanation tonometry, and a visual field examination with Humphrey Field Analyser 3, Carl Zeiss Meditec AG, 24-2 test, full threshold).

The inclusion criteria were the following: (i) normal ophthalmoscopic appearance of the optic disc and peri-papillary retina, (ii) no media opacities, (iii) peri-papillary retinal nerve fiber layer (pRNFL) and visual fields within normal limits, (iv) normal mean IOP on a diurnal tonometric curve (<18 mmHg, mean of three measurements), (v) normal central corneal thickness (CCT), and (vi) BCVA ≥ 20/25.

Exclusion criteria were the following: (i) any ocular disease; (ii) any previous bulbar trauma or intraocular surgery, including retinal photocoagulation laser; (iii) history of IOP > 21 mmHg, spherical refraction ≥ 3.0 D, and cylinder correction ≥2.0 D; (iv) any systemic disease or medication potentially affecting the ONH and peri-papillary retina vascular networks and structures; (v) absence of first-degree familiarity for optic nerve diseases; (vi) any ocular medication administered in the last week. One randomly selected eye of each subject was imaged.

The main outcome measures were pRNFL thickness, vessel density (VD), and flux index (FI) of radial peripapillary capillaries (RPC). The analysis was performed by two observers (D.G., M.T.) independently, in a masked fashion.

### 2.1. Imaging with Optical Microangiography-Based Optical Coherence Tomography Angiography 

**Figure 1:**
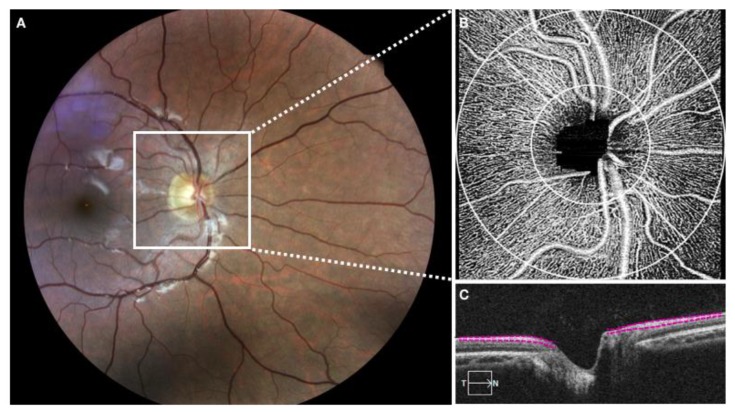
(**A**) Optic nerve head (ONH) and radial peripapillary capillary (RPC) perfusion assessment of healthy eyes. The retinal nerve fibre layer (RNFL) thickness was considered as the distance between the vitreoretinal interface and the ganglion cell layer. (**B**,**C**) A 1.2 mm circular annulus around the ONH (from the outer border of Bruch membrane) was considered for radial peripapillary capillaries assessment.

Pupils were dilated with a combination of 1% tropicamide and 2.5% phenylephrine eye drops. All study participants underwent OCTA imaging using a 68 kHz Cirrus 5000 HD OCT prototype system (wavelength at 840 nm) with active motion-tracking software (Carl Zeiss Meditec Inc, Dublin, CA, USA). An Optic Disc Cube 200 × 200 raster cube OCT scan was acquired using the same device at the time points to assess the global average pRNFL thickness. RNFL thickness was considered as the distance between the vitreoretinal interface and the ganglion cell layer. A 1.2 mm circular annulus around the ONH (from the outer border of Bruch membrane, Figure 1) was considered for RPC assessment. If the boundaries were not correctly delineated, a manual correction was performed by the two experienced observers.

All the acquired volumetric scans were processed with a wavelength at 840 nm, axial resolution of 5 μm, and lateral resolution of 15 μm, with a set of 4.5 × 4.5 mm scans centered on the ONH, 350 × 350 pixels (2× averaged) in the transverse dimension. An internal fixation light was used to centre the scanning area. If the signal strength was below the cutoff (<6), the scans were considered of poor quality and were excluded from the analysis. In order to avoid motion artefacts, an active motion-tracking system of the device was used. VD is defined as the area occupied by vessels in a certain region, and was calculated as a ratio of vessel area within ONH and the whole enface image of ONH area. The flux was normalized to avoid bias, thus calculating only the flux in the vessels. The FI was divided by the full dynamic range of blood flow signal intensity, and was considered as a ratio.

The details of the OMAG algorithm and processing have been already described elsewhere [9,10]. Briefly, OMAG generates three-dimensional, microscopic resolution structural images and vasculature images by identifying differences in the scattered light, due to movements of red blood cells in the vessels, between consecutive B-scans at the same transversal location in the RCP [9].

The coefficients of repeatability (CRs), within subject coefficient of variation (CVw) and intrasession correlation coefficient (ICC), were calculated to evaluate intrasession repeatability of measurements for each observer. Repeatability was tested by means of CVw, ICC, and CR. Intrasession and intersession reproducibility were evaluated by means of a concordance correlation coefficient (CCC). The CCC evaluated the degree to which pairs of observations fall on the 45° line through the origin. It contains a measurements of precision (*p*) that measures how far each observation deviates from the positive bisector and accuracy (*Cb*), which express how far the best-fit line deviates from the 45° line through the origin: *p* = *Cb·p*. The CR is the value below which the absolute differences between two measurements would lie with 0.95 probability; it is directly related to the 95% limits of agreement proposed by Bland–Altman, which contain 95% of the differences between repeated measurements on the same subjects.

### 2.2. Sample Size and Statistical Analysis

Assuming a within-subject standard deviation of 8% and three measurements per subject by two observers using Bland formula, the sample size required to estimate the width of a 95% CI within 8% was 40. The Shapiro–Wilk test was performed to test the normal distribution of the variables. Intrasession repeatability for each observer was measured using two measurements (T_0_ and T_1_). The intra-observer repeatability was evaluated by calculation CR, which is the value below which the absolute differences between two measurements would lie with 0.95 probability; this is directly related to the 95% limits of agreement proposed by Bland–Altman, which contain 95% of the differences between repeated measurements on the same subjects. To quantify the agreement between measurements from T_0_ to T_1_, the Bland–Altman plots was used to calculate the mean differences and constructing limits of agreement for each single examiner. Moreover, repeatability was tested by means of CVw and Lin’s ICC. Intra-observer reproducibility was evaluated with the two-way mixed ANOVA, with the subjects as a random effect and the observers as the fixed effect. In addition, reproducibility was assessed by the CCC, which evaluated the degree to which pairs of observations fall on the 45° line through the origin. It contains a measurement of precision (*p*), which measures how far each observation deviates from the positive bisector, and accuracy (*Cb*), which expresses how the far the best-fit line deviates from 45° line through the origin: *p* = *Cb·p*. Each index used to evaluate repeatability and reproducibility is reported at 95%. To identify outlier measurements before carrying out the Bland–Altman analysis and repeatability coefficients, we identified and excluded data for which the difference was >3 and which were <3 standard deviations (SDs) away from the variable’s mean difference. Statistical analysis was performed using MedCalc statistical software version 18.11.

## 3. Results

A total of 40 healthy subjects (12 males and 28 females) aged 32.5 ± 11.1 years underwent evaluation of ONH-related parameters at baseline (T_0_), after 30 min (T_1_), and after 7 days (T_2_). Demographic characteristics of the people enrolled in this observational study are reported in Table 1. Two patients were excluded from the analysis because of outliers for the variable FI with both observers.

The mean and the standard deviation (SD) of each variable evaluated for each observer over time are described in Table 2.

No statistically significant difference was found among the three different time points for each variable (*p* = 0.427, *p* = 0.103, and *p* = 0.765 for pRNFL thickness, VD, and FI, respectively). The estimate of the effect of the observer, which indicates the bias between observers, was not statistically significant for each variable at all time points (*p* = 0.971, *p* = 0.220, and *p* = 0.157 for pRNFL thickness, VD, and FI, respectively). Moreover, the interaction term for every mixed ANOVA was not statistically significant. A CR, ICC, and CVw were applied to evaluate intrasession repeatability of both two observer measurements in terms of FI, VD, and pRNFL thickness, which is reported in Table 3.

Overall, the agreement between the two observers was excellent (Table 4). In detail, the CCC between the readers was excellent for the pRNFL thickness at each time point; it was also excellent at T_2_ for VD (CCC ≥ 0.8), and was very good for FI and VD at the other time points (Table 4).

The interobserver intrasession reproducibility assessed for T_0_–T_2_ in the variable measurements from the two observers was very good, especially for pRNFL thickness (CCC = 0.956 (95% CI: 0919–0.976) and CCC = 0.961 (95% CI: 0.927–0.979) for Observers 1 and 2, respectively).

Overall, a high CCC was found for measurements of T_0_ and T_2_ time points (CCC ≥ 0.8).

**Figure 2:**
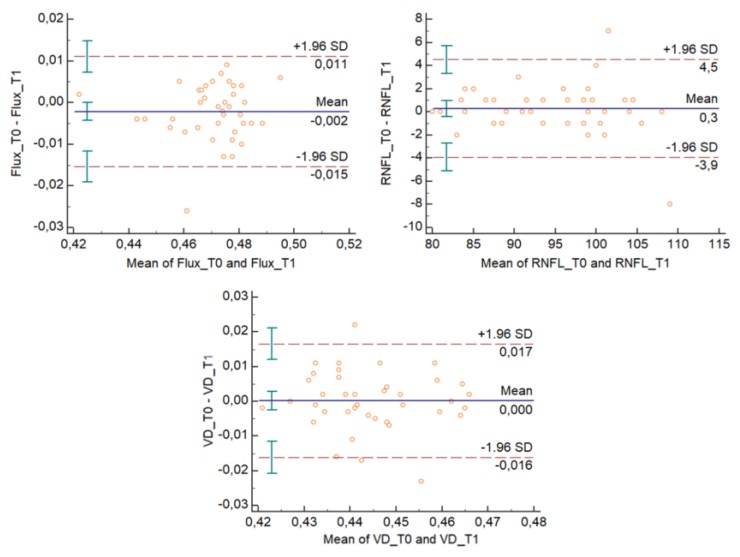
Bland–Altman plots for the flux index (FI), vessel density (VD), and RNFL thickness (RNLF) variables evaluated from T0 to T1, for the first observer. T0 represents measurements by the observer during the first session; T1 represents repeated measurements by the same observer during the same session.

**Figure 3:**
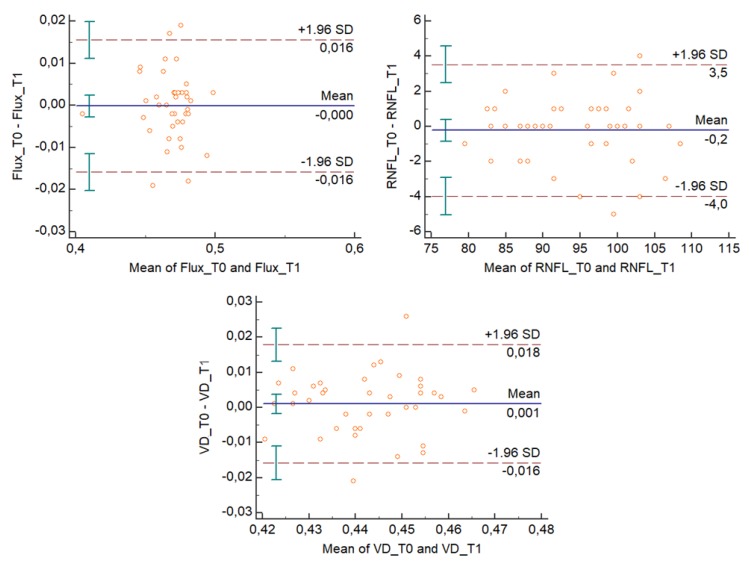
Bland–Altman plots for the flux index (FI), vessel density (VD), and RNFL (RNLF) thickness variables evaluated from T0 to T1, for the second observer. T0 represents measurements by the observer during the first session; T1 represents repeated measurements by the same observer during the same session.

The Bland–Altman plots (Figure 2 and Figure 3) describe the agreement between measurements [11] and represent every difference between the two variables against the average of the measurements. In Figure 2 and Figure 3, the Bland–Altman plots show that for each outcome variable, except for one unit per variable, there is no consistent bias of one observer over the other.

## 4. Discussion

Ocular blood flow changes have been considered to play a key role in the onset and development of different retinovascular and optic nerve diseases [12]. Different retinal imaging techniques have been proposed over the past decades to study ocular blood flow parameters. However, a high range of variability of the coefficients of variation (CVs), which have ranged from 9.7% to 19.8%, and low inter-visit repeatability of these imaging tools have been found [3,4].

The introduction of OCTA has shown promising capability to detect retinal and ONH blood flow in the vessels from the static tissue by performing repeated B-scans located at the same retinal location [13]. It has been previously reported that ONH perfusion is lower in eyes with open-angle glaucoma, compared to healthy eyes [9]. A significant reduction in ONH perfusion and peri-papillary retina was also found in patients with normal tension glaucoma or chronic, non-arteritic, anterior ischemic optic neuropathy [13].

The OMAG technique allows the visualization of microcirculation and is proportional to the blood flow concentration [10]. The results of Chen and coworkers are in line with our findings, since they reported that OMAG has excellent repeatability and reproducibility in identifying and quantifying ONH perfusion in healthy eyes [7,8]. However, those studies were conducted on a very small sample (10 eyes), with all scans performed by the same operator. Furthermore, scans were reviewed by two different observers, which represents a limit in terms of reproducibility. In contrast, we independently assessed the accuracy and reliability of OCTA in the measurements of ONH structural and functional parameters with two different experienced observers, in different sessions and in a masked fashion. Moreover, the three scans of each eye were assessed by each observer at three different moments, in order to avoid daily fluctuations of ONH perfusion.

In addition, the pRNFL thickness was measured with the same device and as previously described [14]. Conversely, Jesus et al. evaluated pRNFL thickness using a Cirrus 5000 HD-OCT by performing all acquisitions with the same operator [14].

Our results showed very good intra-visit repeatability for assessing quantitative and qualitative vascular parameters of the ONH for both operators, with a low CVw (values ranging from 0.535 to 2.017). To be more specific, the smaller the CVw was, the better the repeatability.

Generally, a high CCC means high repeatability, and we found excellent agreement between observer measurements (with values ranging from 0.694 to 0.961). The highest CCC value was found for the pRNFL assessment, most probably because of the absence of diurnal fluctuation for a structural parameter, differently from vascular parameters. Moreover, the OMAG platform requires precise removal of bulk motion by resolving the Doppler phase shift, due to the high sensitivity of the device, which is very susceptible to artifacts from the system or biological phase instability [15].

OCTA is able to quantify, in vivo and in a non-invasive manner, the functional and anatomical features of the retina and ONH. It has great potential for the accurate and reproducible assessment of ONH microcirculation, allowing for repeated measurements without multiple, scan-related sources of bias, thus guaranteeing an operator-independent analysis. An OMAG-based OCTA system has demonstrated itself to be a valuable imaging tool for mapping ONH circulation, with excellent reproducibility and repeatability [9]. Bojikian et al. reported a CVw ≤ 3.7% and ICC ≥ 0.987 for intra-observer and inter-observer data, respectively, in the evaluation of 28 healthy eyes, 30 eyes with open angle glaucoma, and 31 eyes with a diagnosis of normal tension glaucoma [9].

The main limitation of our study was the lack of diseased eyes, to better understand if such absence of disease may affect the accuracy of the device or if some discrepancies in measurements between different operators can be found due to disease-related vascular changes. A recent study has reported that after a circumpapillary microvascular density evaluation, healthy eyes had a higher variation of vascular density, especially in the superior and inferior sectors, if compared to glaucomatous subjects. Indeed, eyes with glaucoma had more uniform vasculature profile, probably related to the typical damages in glaucoma, lowering the whole microvasculature density [14]. Another limitation of our study is that we did not divide ONH in layers or sectors, but we reported vessel data related to the RCP.

A larger sample of eyes and a further sub-analysis considering various ocular diseases would definitely give new insight into the value of OCTA use in clinical practice, as well as its implementation in primary clinical settings. A better comprehension of possible predictive signs on OCTA images to be considered as early disease biomarkers, allowing for prompt diagnosis and treatment, is still a real challenge, especially if vascular damages could precede anatomical modifications of the disc. Further prospective analyses are needed to strengthen these preliminary optimistic data.

## 5. Conclusions

In conclusion, OCTA has demonstrated a great potential in providing an accurate assessment of ONH microcirculation in normal eyes. In particular, the present study shows that this platform allows for repeated and reproducible measurements without multiple scan-related biases, thus guaranteeing an operator-independent analysis with good reproducibility and repeatability. These results show a new potential use of OCTA in ONH vascular assessment for chronic optic nerve “diseases”. This platform may offer a significant clinical utility in assessing such chronic optic nerve diseases.”

## Figures and Tables

**Table 1:** Patients characteristics.

Variable	
Age (years)	32.5 ± 11.08
Gender	
Female	28 (70.0)
Male	12 (30.0)
Eye	
Right	23 (57.5)
Left	17 (42.5)
IOP (mmHg)	16.1 ± 1.4
CCT (µm)	548.2 ± 12.6
Visual field mean deviation (MD: dB)	+0.6 ± 0.8

Data are presented as mean ± standard deviation (SD) or *n* (%).

**Table 2:** Descriptive statistics for each observer at three time points, as well as the overall measurement.

Variables	Observers	T_0_	T_1_	T_2_	Overall
Flux index	Observer 1	0.469 ± 0.014	0.472 ± 0.013	0.471 ± 0.014	0.471 ± 0.014
	Observer 2	0.469 ± 0.016	0.469 ± 0.016	0.470 ± 0.014	0.469 ± 0.015
Vessel density	Observer 1	0.445 ± 0.012	0.444 ± 0.012	0.444 ± 0.013	0.444 ± 0.012
	Observer 2	0.443 ± 0.013	0.442 ± 0.012	0.443 ± 0.014	0.443 ± 0.013
pRNFL (µm)	Observer 1	94.72 ± 7.616	94.43 ± 7.905	94.78 ± 8.042	94.64 ± 7.858
	Observer 2	94.45 ± 7.642	94.68 ± 7.741	94.60 ± 7.405	94.56 ± 7.598

Data are expressed as mean ± standard deviation, pRNFL: peri-papillary retinal nerve fibre layer.

**Table 3:** Coefficient of repeatability (CR), within-subject coefficient of variation (CVw), and intraclass correlation coefficient (ICC). Each index explains the repeatability of measurement for each examiner from T_0_ to T_1_.

Variables	Observer	CR (95% CI)	ICC (95% CI)	CVw (%) (95% CI)
Flux index	Observer 1	0.011 (0.009 to 0.014)	0.870 (0.762 to 0.930)	1.051 (0.535 to 1.387)
	Observer 2	0.016 (0.013 to 0.020)	0.879 (0.783 to 0.934)	1.192 (0.830 to 1.466)
Vessel density	Observer 1	0.016 (0.013 to 0.021)	0.767 (0.600 to 0.870)	1.313 (0.867 to 1.643)
	Observer 2	0.017(0.014 to 0.021)	0.762 (0.595 to 0.867)	1.361 (0.916 to 1.693)
pRNFL (µm)	Observer 1	2.400 (1.948 to 3.092)	0.963 (0.931 to 0.980)	1.508 (0.695 to 2.017)
	Observer 2	3.732 (3.064 to 4.775)	0.970 (0.945 to 0.984)	1.392 (0.999 to 1.696)

A good intrasession repeatability was assessed by the two observers for all three outcome measures (Table 3). The concordance correlation coefficient (CCC) to assess interobserver intrasession reproducibility is reported in Table 4.

**Table 4:** Reproducibility of variables between observers evaluated with concordance correlation coefficient (CCC) at each time point.

		CCC (Observer 1 vs. Observer 2)	95% CI
Flux index	T_0_	0.7790	0.6253 to 0.8746
	T_1_	0.7806	0.6345 to 0.8728
	T_2_	0.7534	0.5816 to 0.8609
Vessel density	T_0_	0.7696	0.6075 to 0.8702
	T_1_	0.6590	0.4445 to 0.8020
	T_2_	0.8891	0.8034 to 0.9388
pRNFL (µm)	T_0_	0.9727	0.9492 to 0.9855
	T_1_	0.9747	0.9529 to 0.9865
	T_2_	0.9688	0.9440 to 0.9828

## References

[1] Lisboa R., Leite M.T., Zangwill L.M., Tafreshi A., Weinreb R.N., Medeiros F.A. (2012). Diagnosing preperimetric glaucoma with spectral domain optical coherence tomography. Ophthalmology.

[2] Zhang A., Wang R.K. (2015). Feature space optical coherence tomography based micro-angiography. Biomed. Opt. Express.

[3] Yousefi S., Wang R.K. (2014). Simultaneous estimation of bidirectional particle flow and relative flux using MUSIC-OCT: Phantom studies. Phys. Med. Biol..

[4] Meira-Freitas D., Lisboa R., Tatham A., Zangwill L.M., Weinreb R.N., Girkin C.A., Liebmann J.M., Medeiros F.A. (2013). Predicting progression in glaucoma suspects with longitudinal estimates of retinal ganglion cell counts. Investig. Ophthalmol. Vis. Sci..

[5] Wang X., Jiang C., Ko T., Kong X., Yu X., Min W., Shi G., Sun X. (2015). Correlation between optic disc perfusion and glaucomatous severity in patients with open angle glaucoma: An optical coherence tomography angiography study. Graefes Arch. Clin. Exp. Ophthalmol..

[6] Hwang J.C., Konduru R., Zhang X., Tan O., A Francis B., Varma R., Sehi M., Greenfield D.S., Sadda S.R., Huang D. (2012). Relationship among visual field, blood flow, and neural structure measurements in glaucoma. Investig. Ophthalmol. Vis. Sci..

[7] Chen C.-L., Zhang A., Bojikian K.D., Wen J.C., Zhang Q., Xin C., Mudumbai R.C., Johnstone M.A., Chen P.P., Wang R.K. (2016). Peripapillary Retinal Nerve Fiber Layer Vascular Microcirculation in Glaucoma Using Optical Coherence Tomography–Based Microangiography. Investig. Ophthalmol. Vis. Sci..

[8] Chen C.-L., Bojikian K.D., Xin C., Wen J.C., Gupta D., Zhang Q., Mudumbai R.C., Johnstone M.A., Chen P.P., Wang R.K. (2016). Repeatability and reproducibility of optic nerve head perfusion measurements using optical coherence tomography angiography. J. Biomed. Opt..

[9] Bojikian K.D., Chen C.L., Wen J.C., Zhang Q., Xin C., Gupta D., Mudumbai R.C., Johnstone M.A., Wang R.K., Chen P.P. (2016). Optic Disc Perfusion in Primary Open Angle and Normal Tension Glaucoma Eyes Using OpticalCoherence Tomography-Based Microangiography. PLoS ONE.

[10] Arend O., Remky A., Plange N., Martin B.J., Harris A. (2002). Capillary density and retinal diameter measurements and their impact on altered retinal circulation in glaucoma: A digital fluorescein angiographic study. Br. J. Ophthalmol..

[11] Bland J.M., Altman D.G. (1999). Measuring agreement in method comparison studies. Stat. Methods Med. Res..

[12] Mastropasqua R., Fasanella V., Agnifili L., Fresina M., Di Staso S., Di Gregorio A., Marchini G., Ciancaglini M. (2015). Advance in the pathogenesis and treatment of normal-tension glaucoma. Prog. Brain Res..

[13] Mastropasqua R., Agnifili L., Borrelli E., Fasanella V., Brescia L., Di Antonio L., Mastropasqua L. (2018). Optical Coherence Tomography Angiography of the Peripapillary Retina in Normal-Tension Glaucoma and Chronic Nonarteritic Anterior Ischemic Optic Neuropathy. Curr. Eye Res..

[14] Jesus D.A., Barbosa Breda J., Van Keer K., Rocha Sousa A., Abegão Pinto L., Stalmans I. (2019). Quantitative automated circumpapillary microvascular density measurements: A new angioOCT-based methodology. Eye (Lond.).

[15] An L., Subhush H.M., Wilson D.J., Wang R.K. (2010). High-resolution wide-field imaging of retinal and choroidal blood perfusion with optical microangiography. J. Biomed. Opt..

